# Double Mutant Analysis with the Large Flower Mutant, *ohbana1*, to Explore the Regulatory Network Controlling the Flower and Seed Sizes in *Arabidopsis thaliana*

**DOI:** 10.3390/plants10091881

**Published:** 2021-09-10

**Authors:** Vuong Quoc Nhat, Yusuke Kazama, Kotaro Ishii, Sumie Ohbu, Hisato Kunitake, Tomoko Abe, Tomonari Hirano

**Affiliations:** 1 Faculty of Agriculture, University of Miyazaki, 1-1 Gakuen-Kibanadai Nishi, Miyazaki 889-2192, Japan; quocnhatvuong@gmail.com (V.Q.N.); hkuni@cc.miyazaki-u.ac.jp (H.K.); 2 Faculty of Bioscience and Biotechnology, Fukui Prefectural University, 4-1-1 Kenjojima, Matsuoka, Eiheiji-cho, Yoshida-gun, Fukui 910-1195, Japan; ykaze@fpu.ac.jp; 3 Nishina Center for Accelerator-Based Science, RIKEN, 2-1 Hirosawa, Wako 351-0198, Japan; ishii.kotaro@qst.go.jp (K.I.); ohbu@riken.jp (S.O.); tomoabe@riken.jp (T.A.)

**Keywords:** argon ion beam, floral organ, mutagenesis, organ size, petal, seed

## Abstract

Two growth processes, cell proliferation and expansion, determine plant species-specific organ sizes. A large flower mutant in *Arabidopsis thaliana*, *ohbana1* (*ohb1*), was isolated from a mutant library. In the *ohb1* flowers, post-mitotic cell expansion and endoreduplication of nuclear DNA were promoted. The whole-genome resequencing and genetic analysis results showed that the loss of function in *MEDIATOR16* (*MED16*), a mediator complex subunit, was responsible for the large flower phenotypes exhibited by *ohb1*. A phenotypic analysis of the mutant alleles in *MED16* and the double mutants created by crossing *ohb1* with representative large flower mutants revealed that MED16 and MED25 share part of the negative petal size regulatory pathways. Furthermore, the double mutant analyses suggested that there were genetically independent pathways leading to cell size restrictions in the floral organs which were not related to the MED complex. Several double mutants also formed larger and heavier seeds than the wild type and single mutant plants, which indicated that MED16 was involved in seed size regulation. This study has revealed part of the size-regulatory network in flowers and seeds through analysis of the *ohb1* mutant, and that the size-regulation pathways are partially different between floral organs and seeds.

## 1. Introduction

The development of plant organs is based on two distinct processes: cell proliferation and expansion, which increase cell number and size, respectively [1], and the regulation of cell proliferation and expansion during morphogenesis contributes to species-specific organ sizes [2]. In floral organs, positive and negative regulatory genes affect each developmental process. The former increases floral organ size, but the latter limits it. During the cell proliferation phase, positive genes, such as *AINTEGUMENTA* [3,4] and *JAGGED* [5], promote cell division, whereas negative genes, including *BIG BROTHER* (*BB*) [6] and *DA1* [7], restrict organ size by limiting cell division. During the post-mitotic cell expansion period, *ROTUNDIFOLIA3* [8,9] and *ARGOS-LIKE* [10] are positive genes that promote cell expansion, whereas *AUXIN RESPONSE FACTOR8* (*ARF8*) [11] and *BIGPETAL* (*BPE*) [12] are negative genes that have been reported to control organ growth by restricting cell size.

In floral organ size regulation, the loss of function of the negative regulatory genes results in large flower phenotypes. The genes are classified broadly into three groups: the ubiquitin pathway, phytohormone signaling pathway, and mediators. It has been reported that ubiquitin-based cell signaling mechanisms are involved in floral organ size regulation. The E3 ubiquitin ligase, BB, and the ubiquitin receptor, DA1, have been found to be negative regulators of floral organ size because they restricted cell proliferation and synergistically acted on size regulation [6,7]. The DA1 also acts synergistically with DA2, a Really Interesting New Gene (RING)-type protein with E3 ubiquitin ligase activity, to negatively regulate floral organ size [13]. A previous study suggested that DA2 and BB/ENHANCER OF DA1 act in different pathways to regulate flower size [13], and that multiple pathways exist that use ubiquitin-based cell signaling to negatively regulate the sizes of floral organs.

Phytohormone signaling pathways also play a role in controlling final floral organ size. BIGPETALp (BPEp) is a basic helix–loop–helix transcription factor which is preferentially expressed in petals and regulates petal growth by restricting post-mitotic cell expansion [12]. It has been demonstrated that *BPEp* expression is regulated by jasmonate signaling and acts downstream of the third isoform of the 12-oxophytodienoic acid reductase (OPR3) involved in jasmonic acid synthesis to control post-mitotic cell expansion and petal growth [14]. Furthermore, AUXIN RESPONSE FACTOR8 (ARF8), which plays a key role in regulating the expression of auxin response genes [15], interacts with BPEp to influence post-mitotic cell expansion during petal development [11]. It has been suggested that the auxin and jasmonate signaling pathway limitation effects on post-mitotic cell expansion during late flower development converge through the interaction between ARF8 and BPEp. The ARF8 also affects cell proliferation during petal development. In addition, ethylene plays a crucial role in organ size regulation [16,17]. The *ethylene insensitve2* (*ein2*) mutant enlarges the size of organs, such as petals, leaves, and stems in *Arabidopsis thaliana*, by increasing the cell size [17]. In *Rosa hybrida*, RhNAC100 transcripts were modulated by ethylene, and silencing the gene in rose flowers led to significantly larger petals because post-mitotic cell expansion had been promoted [16]. RhNAC100 is also known to be a homolog of NAC092/AtNAC02 in Arabidopsis, and NAC092 is regulated by EIN2 in plants [16,18]. These studies have showed that the ethylene signaling pathway regulates post-mitotic cell expansion in a negative way.

*MEDIATOR25* (*MED25*) encodes one of the subunits in the mediator complex, which constructs a molecular bridge to facilitate the interaction between transcription factors (TFs) and RNA polymerase II during transcription [19,20,21,22]. It has been reported that MED25 restricts both cell proliferation and post-mitotic cell expansion, and that *med25* mutants have large floral organs [23]. Therefore, the transcriptional machinery constitutes an important point of regulation in plant organ size control. MED8 and MED16 may also have effects on the relationship between mediator subunits and floral organ size regulation in Arabidopsis. MED8 promotes organ growth via post-mitotic cell expansion, and a phenotype analysis of the *med8 med25* double mutant suggested that MED8 and MED25 do not have a common cell size regulatory pathway, and probably interact with different TFs [24]. It has also been reported that MED16 interacts with MED25, and that a *med16* mutant produced large flower organs through post-mitotic cell expansion [25,26]. MED16 controls endoreduplication and post-mitotic cell expansion in Arabidopsis, including floral organs [26]. However, the details about the relationship between MED16 and the factors for floral organ size regulation have not been fully elucidated.

In Arabidopsis seed size regulation, ovule integuments and seed coat are strongly influenced in a maternal way, and the developmental process of zygotic tissues is also important in determining the seed size. Complicated networks are formed by multiple regulatory pathways, such as cell proliferation and cell expansion during seed coat development, genomic imprinting and parent-of-origin effects in the central cell and endosperm, and endosperm cellularization (reviewed in [27,28]). Phytohormone pathways coordinate seed development and are involved in size determination [28,29]. Many key regulators in the pathways have been identified, and some negative regulators are functionally shared between the regulation of flower size and that of seed size. The factors involved in the ubiquitin pathway described above, BB/EOD1, DA1, and DA2, also control seed size by negatively regulating cell proliferation in maternal tissues [7,13]. Other than the ubiquitin pathway, there is no systematic discussion on the relationship between the negative regulatory pathways of flower size and seed size.

In this study, an Arabidopsis large flower mutant, *ohbana1* (*ohb1*), was isolated from a mutant library induced by heavy-ion beam irradiation. Ionizing radiation has long been used to investigate gene functions, as a mutagen for genetic analysis, and in plant breeding. Recently, heavy-ion beams have also been used as an effective mutagen. They can induce a wide range of mutation phenotypes with high frequencies at relatively low radiation doses compared to traditional mutagens, such as X-rays and γ-rays [30,31], and have been used to analyze gene functions in plants [32,33,34,35,36,37]. In Arabidopsis, heavy-ion beams with different radiation qualities have been used to induce different mutation spectra, especially those based on the size of the deletion mutation [38,39,40]. In addition, heavy-ion beam irradiation of Arabidopsis has been used to produce mutant libraries with different features [39,40,41,42].

This study showed that *MED16* was the gene responsible for the large floral organ phenotype in the *ohb1* mutant by resequencing the mutant genome and genetically analyzing the mutant. Furthermore, the genetic relationships between *MED16* and other floral-size regulation genes were investigated by constructing and analyzing several double-mutant crosses between *ohb1* and different types of representative large flower mutants, including ones that increased petal cell numbers by the ubiquitin pathway (*bb* and *da1*) and others that increased petal cell size by the phytohormone signaling pathway (*arf8*, *bpe*, *ein2*, and *opr3*) and mediator (*med25*). Moreover, we studied whether the negative factors in flower size regulation participate in the seed size regulation.

## 2. Results

### 2.1. Detection of Mutations Induced by Ar Ion Beam Irradiation

**Figure 1:**
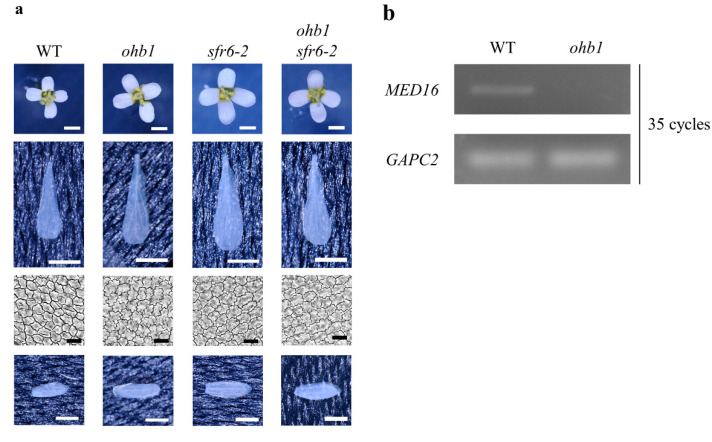
Characteristics of *ohb1* mutant. (**a**) Flower phenotypes of the WT, *ohb1*, *sfr6-2*, and *ohb1 sfr6-2* plants. The whole flowers, petals, petal abaxial epidermal cells, and sepals in each line are shown. White and black bars represent 1 mm and 20 μm, respectively. (**b**) RT-PCR analysis of MED16 expression in WT and *ohb1*. The transcript amount of *GCP2* was used as an internal control in the RT-PCR.

The *Ar50-46-pl1* mutant line was screened from a mutant library induced by Ar-ion beam irradiation at the RIKEN Nishina Center [40,41]. It had a large flower phenotype (Figure 1a) and was named *ohbana1* (*ohb1*), meaning “Large flower” in Japanese. A whole-genome mutation analysis was performed to detect candidate genes responsible for the large flower phenotype. In the M_3_ generation, five homogenous mutations in three loci, which had been predicted to affect gene function, were detected in the mutant genome (Table 1). Therefore, three mutated genes were candidates responsible for the mutant phenotype. In linkage analyses after backcrossing, a total of 23 plants out of 126 plants showed a large flower phenotype and homozygous mutations in AT4G04920 (Table S1), which was previously characterized as *MED16* [25,26]. Reverse transcription PCR (RT-PCR) analysis was applied to confirm the gene expression of *MED16* in *ohb1*. The expression of *MED16* was barely detected in *ohb1* (Figure 1b). These results indicated that the recessive mutated gene in AT4G04920 could be responsible for the large flower phenotype and that *ohb1* is a null mutant of *MED16*.

### 2.2. Characteristics of the Floral Organs in ohb1

The phenotypic characteristics of the *ohb1* mutant were evaluated by comparing *ohb1* sepal and petal sizes to those produced by wild type (WT) flowers. The *ohb1* mutant formed large floral organs (Figure 1a), in which the length and width of the petals and sepals were approximately 1.2 times larger than those in WT flowers (Table 2). Furthermore, in the *ohb1* mutant, the relative value of the petal area was 142, which was similar to that of the abaxial epidermal cells in the petals (145) (Table 2). In the complementation test, *ohb1* was crossed with *sensitive to freezing6* (*sfr6*)-*2*, in which T-DNA was inserted into AT4G04920 [43]. Then, the floral organ sizes in the F_1_ generation (*ohb1 sfr6-2*) were analyzed. The results showed that the organ sizes in *ohb1 sfr6-2* were almost the same as those in *ohb1* (Table 2), which showed that the mutation in *med16*/*sfr6* was responsible for the floral organ phenotypes exhibited by *ohb1*. 

**Figure 2:**
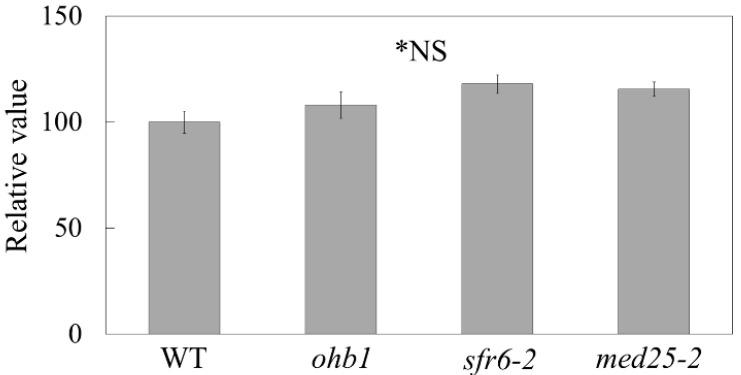
Total number of the petal epidermal cells in the WT and each large flower mutant. Each value is expressed as the mean ± SE relative to the respective WT value, which was set at 100. * There is no significant difference between the lines according to Tukey’s test (n = 16, *p* < 0.05).

The total number of the petal epidermal cells was analyzed to evaluate contributions made by MED16 to cell proliferation restriction in floral organs (Figure 2). Although the petal epidermal cells tend to increase in *ohb1*, *sfr6-2*, and *med25-2* [23] compared with WT, there was no significant difference among all lines tested. These results suggest that the production of large petals by *ohb1* was due to the increase in cell size rather than an increase in cell numbers.

A flow cytometric analysis using the nuclei of whole flowers from WT and *ohb1* was carried out to elucidate whether post-mitotic cell expansion in *ohb1* was caused by endoreduplication. The proportions of 4C and 8C cells in *ohb1* were significantly higher than in WT (Table 3), which indicated that endoreduplication had been promoted in *ohb1* floral organ cells. 

### 2.3. Double-Mutant Crosses between ohb1 and Large Flower Mutants Associated with Post-Mitotic Cell Expansion

**Figure 3:**
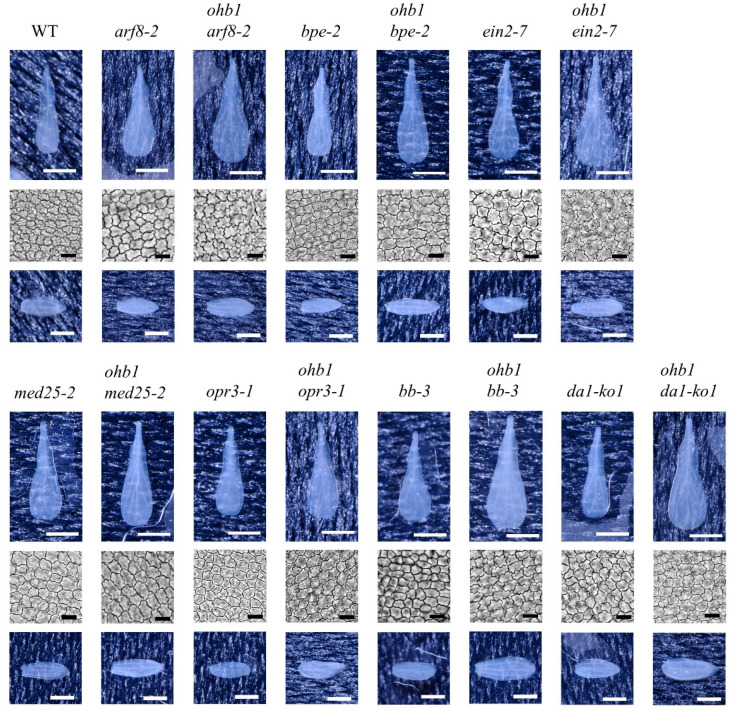
Phenotypic characteristics of flowers produced by previously identified large flower mutants and double mutants that had been created through a cross with the *ohb1* mutant. Petals, petal abaxial epidermal cells, and the sepals in each line are shown. White and black bars represent 1 mm and 20 μm, respectively.

**Figure 4:**
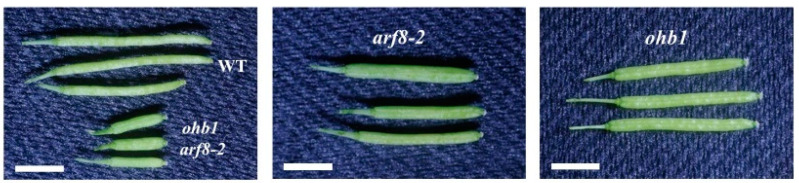
Fruits produced by the mutant lines related to *ohb1* and *arf8*. Bars represent 3 mm.

A total of five mutant lines: *arf8-2* [44], *bpe-2* [12], *ein2-7* [45], *med25-2*, and *opr3-1* [14], which are large flower mutants associated with post-mitotic cell expansion, were used to construct double-mutant lines by crossing them with *ohb1*. The floral organ phenotypes for each single mutant showed similar characteristics to those reported in previous studies (Figure 3, Table 2) [11,12,14,17,23]. A double mutant, *ohb1 arf8-2*, formed larger petals and sepals than each single mutant (Figure 3). The cell size of the petals in the double mutant was almost the same as that in *ohb1* (Table 2). In addition, the fruits produced by the double mutant were smaller than those produced by each single mutant (Figure 4), and the fruit length in the double mutant was reduced to approximately 38% of that in WT. The *ohb1 bpe-2*, *ohb1 ein2-7*, and *ohb1 opr3-1* flowers had larger petals and sepals compared to each single mutant (Figure 3). The *ohb1 med25-2* mutant plants had larger floral organs than *ohb1* (Table 2). However, none of the increasing rates in the *ohb1 med25-2* floral organs were significantly higher than those in the *med25-2* organs (Table 2). 

### 2.4. Double-Mutant Crosses between ohb1 and Large Flower Mutants Associated with Cell Proliferation

The two large flower mutants associated with cell proliferation, *bb-3* [6] and *da1-ko1* [7], were used to construct double-mutant lines. The *bb-3* and *da1-ko1* mutants formed large flowers, and the petals and sepals produced by each mutant were larger than those produced by the WT flowers (Figure 3, Table 2). The abaxial epidermal cells in *bb-3* and *da1-ko1* petals were the same cell size as those in WT (Table 2), and the increase rates for petal area were synergistically enhanced in *ohb1 bb-3* and *ohb1 da1-ko1* (Figure 3). The epidermal cell sizes in *ohb1 bb-3* and *ohb1 da1-ko1* petals were almost the same size as those in *ohb1* petals (Table 2).

### 2.5. Seed Weights Produced by the Large Flower Mutants

**Figure 5:**
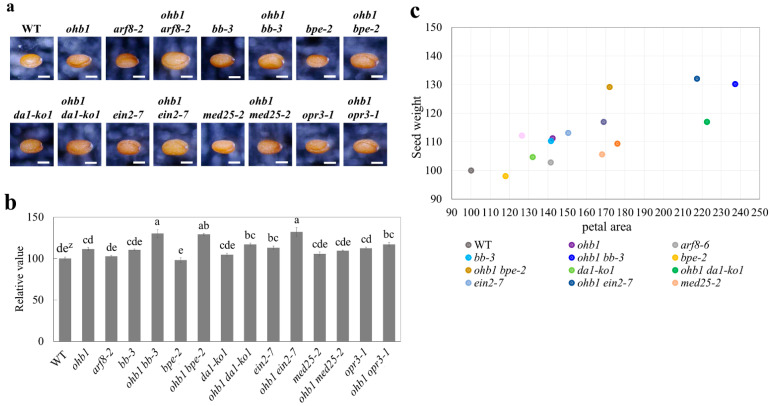
Phenotypic characteristics of the seeds produced by the single and double mutants derived from *ohb1* and previously known large flower mutants. (**a**) Seeds from the WT and each large flower mutant. Bars represent 250 μm. (**b**) Comparison of the seed weights among the 15 lines except for *ohb1 arf8-2*. Each value is expressed as the mean ± SE relative to the WT value, which was set at 100. ^z^ Different letters represent significant differences between mutants according to Tukey’s test (n = 5, *p* < 0.05). (**c**) Scatter plot of petal area versus seed wight.

The seed weights for each large flower mutant line were measured and compared. The seeds produced by the large flower mutants tended to be larger and heavier than the WT seeds (Figure 5a,b). The *ein2-7* single mutant produced the heaviest seeds among the single mutants (Figure 5b). The *ohb1 bb-3*, *ohb1 bpe-2*, and *ohb1 ein2-7* seed weights were enhanced by double mutant formation because the *ohb1 bb-3*, *ohb1 bpe-2*, and *ohb1 ein2-7* seed weights were significantly heavier than those produced by each single mutant. The *ohb1 med25-2* seed sizes and weights were almost the same as the single mutant weights and sizes. It was impossible to collect a sufficient number of seeds from *ohb1 arf8-2* to carry out a seed weight analysis due to the low fertility levels exhibited by this mutant (data not shown). A positive correlation (r = 0.795) was observed between the petal area and the seed weight (Figure 5c).

## 3. Discussion

*MED16* is a gene that encodes one of the mediator subunits, and has been identified as a multifunctional regulator of floral transition, cell wall composition, plant immunity, iron homeostasis, and cold responses [20,21,25,43,46,47,48,49,50,51]. In this study, MED16 was identified as a regulator of floral organ size. In *med16* mutant alleles, *ohb1* and *sfr6-2* were thought to be loss-of-function alleles (Figure 1b) [26], and increased cell size led to the formation of large petals compared to WT flowers (Figure 1, Table 2), which indicated that MED16 restricts post-mitotic cell expansion to control the final flower size in Arabidopsis. These results agree with a previous study [26]. 

In a prior study, MED25 was reported to modulate floral organs by restricting cell size enlargement and proliferation [23]. The MED25 protein is also a mediator complex [19,20] and interacts with MED16 [25]. The phenotype analysis of the *ohb1 med25-2* double mutant suggested that *MED16* and *MED25* partially shared a negative cell size regulatory pathway. MED16 interacts with the transcriptional repressor DP-E2F LIKE1 and controls endoreduplication by repressing the *CELL CYCLE SWITCH52A1*/*A2* genes, which are essential for the transition from mitosis to endoreduplication [25]. The results also showed that there were ploidy level changes in the ohb1 floral organs, and that the 4C and 8C nuclei proportions increased (Table 3). However, it was reported that endoreduplication is not enhanced in *med25-2* [23]. In general, endoreduplication is associated with a cell-size increase [52]. On the other hand, changes in endoreduplication cannot fully explain cell size phenotypes of mutants [53]. Although the cell size increase in *ohb1* could be due to an increase in endoreduplication, other factors possibly regulate the cell size. MED25 is thought to regulate cell size by restricting the expression of expansin genes [23]. The mediator subunits have pleiotropic functions in the size regulation of floral organs via the transcriptional regulation of target genes. Further research is imperative to reveal detailed roles of each mediator subunit during the size regulation of floral organs. It is possible that lignification is one of the factors for the size regulation by the mediator subunits. MED5a/5b are required for both the stunted growth and the lignin deficiency of *reduced epidermal fluorescence 8* mutants in Arabidopsis [54]. 

In *med25-2*, cell number increases slightly contribute to form large petals in comparison to cell size increase [23]. Although the petal epidermal cells tend to increase in *ohb1*, *sfr6-2*, and *med25-2* compared with WT in this study, there is no significant difference between them (Figure 2). It might be difficult to detect the slight increment observed in *med25-2*, by such a calculation. To further reveal mechanisms on the cell proliferation restriction by the mediator subunits, a more detailed analysis is required, considering the developmental stage and petal region.

In addition to *ohb1 med25-2*, the phenotype analysis of the double mutants helped reveal the genetic network controlling floral organ size regulation. In the double mutants produced by crossing *ohb1* with large flower mutants associated with cell proliferation, *ohb1 bb-3* and *ohb1 da1-ko1* showed a remarkable synergistic effect in petal area compared to the single mutants (Table 2), which clearly indicated that the regulatory pathway for cell number is different from that of the cell size in petals. The *ohb1 ein2-7* petal cell area was the largest in the mutant lines and was synergistically increased compared to the single mutants (Table 2). It has been reported that the ethylene signaling pathway regulates post-mitotic cell expansion in a negative way [16,17], and that the MED complex also requires transcriptional regulation of the genes involved in the post-mitotic cell expansion of floral organs. The results from this study suggested that these two regulatory pathways work independently during post-mitotic cell expansion in the floral organs. 

The *ohb1 bpe-2* double mutant increased petal cell size through the independent functions of *ohb1* and *bpe-2*. Furthermore, the double mutant constructed by crossing *ohb1* with *opr3-1* also had large flowers that had increased in an additive manner. The correlation between *ohb1* and the jasmonate pathway is generally consistent because the BPEp acts downstream of OPR3, which is involved in jasmonic acid synthesis and controls post-mitotic cell expansion in petals [14]. In addition, the increases in the petal cell sizes of the *ohb1 arf8-2* double mutant were almost the same as those for *ohb1* (Table 2). It has been reported that ARF8 interacts physically with BPE to control cell size by restricting post-mitotic expansion and that it also regulates cell proliferation during petal development [11]. Therefore, the difference in the cell areas between *ohb1 arf8-2* and *ohb1 bpe-2* may be due to the effect of the ARF8 function during cell number regulation. It has been reported that there is a relationship between cell proliferation and expansion during organ size control [55], and that *more and smaller cell* mutants exhibit phenotypes with increased cell numbers and decreased cell sizes [56]. It is thought that cell size restriction in *ohb1 arf8-2* compared to *ohb1 bpe-2* is affected by cell number increase.

Several large flower mutants also increased their seed weight compared to the WT, and positive correlation (r = 0.795) was observed between the petal area and the seed weight (Figure 5c). The genes responsible for these mutants, such as *BB* and *DA1*, have been shown to control seed size [6,7]. Mutations in the *ARF2* gene are known to increase seed, flower, and other organ sizes [57,58]. However, it has been reported that *med25* mutants produce larger flowers but the seed size does not increase [23], which supports the results produced by this study (Figure 5). The results from this study indicated that there are partially close relationships that control seed and floral organ size. Although the seeds used in this study were derived from open pollination, the results would show a feature of seed change in the mutants for the most part because of the seeds bulked from many fruits. The *ohb1 ein2-7* double mutant had significantly heavier seed weights than the single mutants (Figure 5), which indicated that *ohb1* and *ein2-7* increased their seed weights in an additive manner. It is possible that *MED16* and *EIN2* are part of the independent pathways that regulate seed size. The *arf8-2* seed weight was almost the same as the WT seed weight (Figure 5b), which suggested that *ARF8* was not associated with seed weight regulation. The *ohb1 arf8-2* mutant formed small fruits and had low fertility levels despite the fact that the single mutants did not produce small fruits and generated a number of seeds (Figure 4). Nagpal et al. [59] reported that the *arf6 arf8* double mutants were female sterile and their stigmatic papillae did not elongate. The results from this study showed that *ohb1 arf8-2* had a similar fertility phenotype to *arf6 arf8*. Therefore, it seems that *MED16* is partially related to the *ARF* genes. In *ohb1 bpe-2*, the seed weight of the double mutant was heavier than that of each single mutant. However, the *bpe-2* single mutant did not increase its seed weight at all compared to the WT (Figure 5b). It is possible that *bpe-2* enhances the *ohb1* effect on seed weight. The *ohb1 opr3* seed weight was smaller than the *ohb1 bpe-2* seed weight, even though post-mitotic cell expansion in petals was similar in *ohb1 bpe-2* and *ohb1 opr3*. It is possible that OPR3 might regulate cell size in seeds via a different pathway from the one operating in flowers. The seed weight results of the *ohb1* double mutants suggest that MED16 may be involved in seed size regulation. It would be deeply interesting to know if the seed changes observed in this study were derived from the developmental processes of the maternal tissue and/or zygotic tissues. A part of the seed changes in the mutants are possibly associated with changes in fertility and seed production, such as the *ohb1 arf8-2* mutant. Detailed studies in the fertility and seed production are required for the mutants. Increasing the mass of seeds is directly linked to high crop yields. Therefore, future studies should use large flower mutants to analyze the relationship between flower size and seed size, and to further understand the regulation of seed development.

Flower size is a very important trait for floriculture plants. The “breeding of large flowers” has been carried out over many years. However, the genetic regulation of floral organ size is not fully understood. The analysis of large flower mutants in this study revealed genetic relationships that are involved in the floral organ size regulation network. *MED16* may act with other negative regulatory genes to control flower and seed size. The double mutant analysis revealed that the *med16* mutation has additive and synergistic enlargement effects on the floral organs produced by plants with large flower mutant backgrounds, and that the size-regulation pathways for floral organs and seeds are partially different. Some mediator subunits, including MED16, have been reported to contribute to floral organ size regulation, and the transcriptional machinery, including the mediator complex, is highly conserved in higher plants. Further studies on the relationships between flower size regulation networks and mediator complexes will be needed if the mechanism controlling flower size regulation is to be elucidated, particularly in non-model plants.

## 4. Materials and Methods

### 4.1. Plant Materials and Growth Conditions

The *A*. *thaliana* accession Columbia-0 (Col-0) was used as the WT, and all mutants used in this study had the Col-0 background. *Ar50-46-pl1* (*ohb1*) and *C30-1-as1* were derived from the heavy-ion-induced mutant libraries [37,38]. *C30-1-as1* was renamed *ein2-7* because *C30-1-as1* harbors a homozygous mutation in *EIN2* [45]. *Ar50-46-pl1* was backcrossed with WT in the phenotypic and linkage analyses. After self-fertilization of the backcrossed plants, *ohb1* mutants were selected based on their phenotype, and then the seeds from the selected mutants were harvested for a phenotypic analysis. A total of seven T-DNA tagged lines were obtained from the Arabidopsis Biological Resource Center as mutant alleles of the negative regulatory genes for floral organ size. These were *arf8-2* (SAIL_17_D08) [44], *bb-3* (SAIL_794_E08) [6], *bpe-2* (SALK_089219) [12], *da1-ko1* (SALK_126092) [7], *med25-2* (SALK_080230) [23], *opr3-1* (SALK_120896) [14], and *sfr6-2* (SALK_048091) [43]. The *sfr6* mutant has a T-DNA insertion at AT4G04920 as one of the candidate genes responsible for the *ohb1* phenotype. The double-mutant lines were constructed by crossing the ohb1 mutant and the T-DNA tagged lines. Seeds from each line were surface-sterilized for 10 min with a sodium hypochlorite solution (1% available chlorine) containing 0.1% polyoxyethylene (20) sorbitan monolaurate. The seeds were washed three times with sterile distilled water and placed on 0.7% (*w*/*v*) agar-solidified 1/2 Murashige–Skoog medium with 3.0% (*w*/*v*) sucrose. Then, they were incubated at 23 °C with a 16 h light/8 h dark cycle. After 3 weeks, the seedlings were moved to plastic pots filled with culture soil and grown in an incubator under the same conditions mentioned above.

### 4.2. Whole-Genome Mutation Analysis

The *Ar50-46-pl1* resequencing process was performed at the Takara Dragon Genomics Center (Takara Bio Inc., Mie, Japan) using a HiSeq 2000 sequencing system (Illumina Inc., San Diego, CA, USA). The leaves used in the genomic DNA isolation process were collected from 40 M_3_ generation mutants. Genomic DNA isolation and library construction were performed according to Hirano et al. [60]. The reads obtained were inputted into the automated mutation analysis pipeline (AMAP) [61] and AMAP execute programs for mapping, the removal of potential PCR duplicates, detection of mutations, and an integrated set of mutation analyses. Nucleotide sequence data files are available in the DDBJ Sequenced Read Archive under the accession number DRA010751. Pre-existent polymorphisms in the WT were evaluated by comparing them to detected mutations in mutants induced by heavy-ion beam radiation [45], and mutation candidates that were detected in at least two mutants were removed as false-positive mutations. The mutations that were predicted to affect gene function are listed in Table 1.

### 4.3. RT-PCR

Total RNA was isolated from WT and ohb1 leaves by NucleoSpin RNA XS kit (MACHEREY-NAGEL, Düren, Germany) following the manufacturer’s instruction. Reverse transcription of the isolated RNA (500 ng) was performed with SuperScript IV First-Strand Synthesis System protocol (Thermo Fisher Scientific, MA, USA). The cDNA (1.0 μL) of each sample was subjected to RT-PCR using PrimeSTAR GXL DNA Polymerase (TaKaRa Bio, Shiga, Japan) and primer sets: forward primer (P1) 5′-TTAGTGCACGACGCTACCTC-3′ and reverse primer (P2) 5′-CATGGCACCACTGTCTGTTA-3′ for *MED16* [25] and forward primer 5′-TTGGTGACAACAGGTCAAGCA-3′ and reverse primer 5′-AAACTTGTCGCTCAATGCAATC-3′ for *glyceraldehyde-3-phosphate dehydrogenase C2* (*GAPC2*).

### 4.4. Morphological Analysis of the Floral Organs

The flowers at stage 14 of floral morphogenesis [62], which had fully expanded petals but had not been pollinated, were collected and placed into 1.5 mL tubes with fixative solution containing 86% ethanol and 14% acetic acid. They were then left to fix overnight at 4 °C. After fixation, the flowers were placed on a Petri dish for dissection using a stereoscopic microscope (SZ2-ILST, Olympus, Tokyo, Japan), and one of the separated petals and sepals from each flower were photographed using a stereoscopic microscope. Then, abaxial epidermal cells in the distal region of the petals were observed and photographed using an optical microscope (BX51TRF, Olympus, Japan). The sizes of the petals (length, width, and area), sepals (length and width), and petal epidermal cells were measured using ImageJ software [63]. Twenty petals and ten sepals were measured in each line during the size analysis. The epidermal cell size in each petal was calculated as the average of 100 cells per petal, and 20 petals in each line were used for the observation. The total number of the petal epidermal cells was estimated by cell number per unit area and the petal area.

Seed weight was analyzed by collecting mature seeds from five individuals of each line. Before measurement, the collected seeds were dried using desiccant agents overnight at room temperature. One hundred dry seeds per individual were counted, and their weights were measured.

Absolute values of the phenotypic data in the flowers and seeds are shown in Table S2.

### 4.5. Flow Cytometric Analysis

The nuclear ploidy level in the floral organs was determined using a flow cytometer (CyFlow counter, Sysmex, Kobe, Japan). One whole flower from each WT and ohb1 plant was chopped up with a sharp razor blade in 200 μL nuclear extraction buffer (CyStain UV precise P kit, Sysmex, Lincolnshire, IL, USA). Then, 800 μL staining buffer from a CyStain UV precise P kit was added to the nuclear extraction buffer. The suspension was filtered through a 30 μm nylon mesh and analyzed using a flow cytometer. The experiment was repeated six times.

### 4.6. Statistical Analysis

The nuclear ploidy levels in the flowers from the WT and *ohb1* mutants were compared using a *t*-test. The mutant value for each phenotypic character was represented as being relative to the WT value, which was set at 100. The relative values were compared using Tukey’s test.

## Figures and Tables

**Table 1:** List of candidate mutations responsible for the large flower phenotype shown by the *ohb1* mutant.

Chr.	Position	Locus	Reference Base	Mutated Base	Effect of Mutation
2	19,044,495	AT2G46400	*	−42 bp	codon change codon deletion
4	2,501,011	AT4G04920	*	+A	frame shift
4	2,501,014	AT4G04920	T	A	non synonymous coding
4	2,501,015	AT4G04920	*	−TTCGG	frame shift
4	5,349,509	AT4G08430	G	A	non synonymous coding

**Table 2:** Phenotypic characteristics of the flowers produced by the large flower mutants and the double-mutant lines created through a cross with *ohb1*.

Line	Petal				Sepal	
	Length	Width	Area	Cell Area	Length	Width
WT	100.0 ± 2.3 ^e^	100.0 ± 2.9 ^e^	100.0 ± 4.5 ^g^	100.0 ± 2.5 ^h^	100.0 ± 3.1 ^g^	100.0 ± 3.5 ^f^
*ohb1*	116.6 ± 2.4 ^d^	119.1 ± 3.6 ^d^	142.3 ± 7.9 ^de^	145.3 ± 2.5 ^def^	119.7 ± 3.7 ^cdef^	125.2 ± 4.4 ^cd^
*sfr6-2*	127.4 ± 1.7 ^bc^	123.5 ± 2.3 ^cd^	159.3 ± 4.5 ^bcd^	157.7 ± 2.4 ^bcd^	122.8 ± 2.2 ^bcde^	121.9 ± 6.1 ^cde^
*ohb1 sfr6-2*	119.7 ± 2.2 ^cd^	119.6 ± 2.4 ^d^	141.4 ± 4.4 ^def^	145.3 ± 3.5 ^def^	123.1 ± 2.8 ^bcde^	124.7 ± 2.8 ^cde^
*arf8-2*	115.0 ± 1.3 ^d^	123.9 ± 2.3 ^cd^	141.4 ± 3.5 ^def^	117.1 ± 1.8 ^g^	109.9 ± 1.9 ^efg^	119.2 ± 5.2 ^cdef^
*ohb1 arf8-2*	134.2 ± 1.6 ^b^	156.0 ± 2.3 ^a^	208.8 ± 5.9 ^a^	147.6 ± 1.5 ^cde^	128.1 ± 3.2 ^bc^	150.2 ± 4.8 ^ab^
*bpe-2*	112.5 ± 1.7 ^d^	104.4 ± 1.6 ^e^	117.9 ± 3.1 ^fg^	118.7 ± 2.0 ^g^	106.7 ± 2.2 ^fg^	103.7 ± 4.1 ^ef^
*ohb1 bpe-2*	133.8 ± 1.7 ^b^	128.2 ± 1.4 b^cd^	171.9 ± 3.3 ^bc^	168.6 ± 2.6 ^b^	133.3 ± 1.9 ^ab^	124.5 ± 3.5 ^cde^
*ein2-7*	126.4 ± 1.8 ^bc^	128.4 ± 2.0 ^bcd^	150.4 ± 4.2 ^cde^	156.8 ± 3.9 ^bcd^	113.3 ± 1.9 ^defg^	120.7 ± 4.7 ^cdef^
*ohb1 ein2-7*	148.2 ± 1.8 ^a^	164.8 ± 2.7 ^a^	217.3 ± 4.7 ^a^	230.8 ± 5.0 ^a^	130.4 ± 1.8 ^abc^	134.1 ± 3.9 ^bcd^
*med25-2*	128.3 ± 1.7 ^bc^	135.6 ± 2.3 ^bc^	168.0 ± 3.3 ^bc^	160.4 ± 2.0 ^bc^	124.4 ± 3.1 ^bcd^	133.0 ± 3.7 ^bcd^
*ohb1 med25-2*	133.2 ± 1.9 ^b^	134.9 ± 1.9 ^bc^	175.9 ± 4.8 ^b^	166.7 ± 1.9 ^b^	126.9 ± 3.2 ^bc^	131.3 ± 3.6 ^bcd^
*opr3-1*	113.1 ± 1.1 ^d^	118.5 ± 1.2 ^d^	126.5 ± 2.4 ^ef^	133.3 ± 2.1 ^f^	106.2 ± 2.0 ^g^	116.7 ± 3.3 ^def^
*ohb1 opr3-1*	126.7 ± 1.9 ^bc^	137.5 ± 2.0 ^b^	168.9 ± 5.0 ^bc^	158.6 ± 2.1 ^bc^	124.7 ± 2.0 ^bcd^	136.0 ± 3.8 ^bcd^
*bb-3*	113.9 ± 1.7 ^d^	134.3 ± 2.2 ^bc^	141.4 ± 3.7 ^def^	103.4 ± 2.6 ^h^	113.4 ± 2.6 ^defg^	139.7 ± 3.5 ^bc^
*ohb1 bb-3*	144.4 ± 2.4 ^a^	170.4 ± 4.2 ^a^	237.2 ± 9.1 ^a^	139.8 ± 2.8 ^ef^	142.0 ± 4.4 ^a^	164.5 ± 5.5 ^a^
*da1-ko1* *ohb1 da1-ko1*	115.9 ± 1.6 ^d^	117.5 ± 2.2 ^d^	132.0 ± 3.5 ^ef^	103.6 ± 1.8 ^h^	112.9 ± 2.2 ^defg^	127.1 ± 4.5 ^cd^
	144.7 ± 1.5 ^a^	158.9 ± 3.1 ^a^	222.5 ± 5.2 ^a^	144.8 ± 2.5 ^def^	134.3 ± 2.4 ^ab^	152.7 ± 4.5 ^ab^

Each value is expressed as the mean ± SE relative to the respective WT value, which was set at 100. Different letters in each column represent significant differences between the mutants according to Tukey’s test (*p* < 0.05, n = 20 and 10 for each petal and sepal category, respectively).

**Table 3:** Proportion of floral organ cells with different nuclear DNA contents calculated by flow cytometry analysis.

Line	2C	4C	8C
WT	72.5 ± 0.9	24.9 ± 0.7	2.6 ± 0.3
*ohb1*	58.8 ± 2.7 *	34.9 ± 2.1 *	6.2 ± 0.7 *

Each value is expressed as the mean ± SE for six individual experiments. * Values in each column are significantly different *(p* < 0.01) according to a *t*-test.

## Data Availability

The data presented in this study are openly available in the DDBJ Sequenced Read Archive, under the accession number DRA010751.

## Supplementary Materials

The following are available online at https://www.mdpi.com/article/10.3390/plants10091881/s1, Table S1: linkage analysis in 23 backcrossed lines with large flower phenotype, Table S2: Absolute values of phenotypic data in the large flower mutants and the double mutant lines created through a cross with *ohb1*.
