# Supplementary material for: Double Mutant Analysis with the Large Flower Mutant, ohbana1, to Explore the Regulatory Network Controlling the Flower and Seed Sizes in Arabidopsis thaliana
Source: Plants (Basel). 2021 Sep 10;10(9):1881. doi: 10.3390/plants10091881 (PMC8473154; doi:10.3390/plants10091881)
Supplement: Supplementary file 1 [file plants-10-01881-s001.zip › plants-1341009-supplementary.pdf]

Table S1 linkage analysis in 23 backcrossed lines with large flower phenotype.

	backcrossed lines with large flower phenotype																						
locus	1	2	3	4	5	6	7	8	10	11	12	13	14	15	16	17	18	19	20	21	22	23	24
AT2G46400[X]	W/H	W/H	M	W/H	W/H	W/H	W/H	W/H	W/H	W/H	W/H	W/H	W/H	W/H	W/H	W/H	W/H	W/H	W/H	W/H	W/H	W/H	W/H
AT4G04920[Y]	M	M	M	M	M	M	M	M	M	M	M	M	M	M	M	M	M	M	M	M	M	M	M
AT4G08430[Z]	M	M	M	M	M	H	M	M	M	H	M	H	H	M	W	M	M	M	M	M	M	M	M

[X]Deletion at AT2G46400 was detected by PCR with primer sets: forward primer 5′ -CAGCAGCAGGTGCAGATAAA-3′ and reverse primer 5′ -TTCTAAAAACGTCTTCAAAAAGAGG-3′ . The reverse primer was designed on the deletion.

[Y]Mutations at AT4G04920 were detected by high resolution melting curve analysis with primer sets: forward primer 5′ -AAGGAGCCTGATTCTGGTGA-3′ and reverse primer 5′ -AGCAGCATCATGCCATACAG-3′ .

[Z]Region including base change at AT4G08430 was amplified with primer sets: forward primer 5′ -TGGCAGCTTCATTGCATACT-3′ and reverse primer 5′ -GTGCGTGTGAGCCATATGAT-3′ . The amplified fragment was sequenced.

W/H：homozygous of WT allele or hetelozygous of WT allele and mutant allele, H: hetelozygous of WT allele and mutant allele, M: homozygous of mutant allele.

Table S2 Absolute values of phenotypic data in the large flower mutants and the double mutant lines created through a cross with *ohb1*

	petal					sepal		
	length (mm)	width (mm)	area (mm^2)	cell area (μm^2)	Total number of epidermal cells	length (mm)	width (mm)	seed weight (mg)
WT	2.36 ± 0.05	0.723 ± 0.021	1.08 ± 0.05	151.5 ± 3.8	5359 ± 275	1.65 ± 0.05	0.575 ± 0.020	2.12 ± 0.04
ohb1	2.75 ± 0.06	0.860 ± 0.026	1.54 ± 0.09	220.1 ± 3.7	5787 ± 326	1.98 ± 0.06	0.720 ± 0.025	2.36 ± 0.04
sfr6-2	3.01 ± 0.04	0.892 ± 0.017	1.72 ± 0.05	238.8 ± 3.6	6319 ± 234	2.03 ± 0.04	0.701 ± 0.035	-
ohb1 sfr6-2	2.83 ± 0.05	0.864 ± 0.018	1.53 ± 0.05	220.0 ± 5.4	5693 ± 183	2.03 ± 0.05	0.717 ± 0.016	-
arf8-2	2.72 ± 0.03	0.895 ± 0.016	1.53 ± 0.04	177.4 ± 2.8	-	1.81 ± 0.03	0.685 ± 0.030	2.18 ± 0.02
ohb1 arf8-2	3.17 ± 0.04	1.127 ± 0.017	2.26 ± 0.06	223.5 ± 2.3	-	2.11 ± 0.05	0.863 ± 0.027	-
bpe-2	2.66 ± 0.04	0.754 ± 0.012	1.27 ± 0.03	179.8 ± 3.0	-	1.78 ± 0.04	0.595 ± 0.026	2.08 ± 0.06
ohb1 bpe-2	3.16 ± 0.04	0.926 ± 0.010	1.86 ± 0.04	255.4 ± 4.0	-	2.20 ± 0.03	0.716 ± 0.020	2.74 ± 0.02
ein2-7	2.99 ± 0.04	0.928 ± 0.014	1.63 ± 0.05	237.5 ± 5.9	-	1.87 ± 0.03	0.694 ± 0.027	2.40 ± 0.04
ohb1 ein2-7	3.50 ± 0.04	1.190 ± 0.019	2.35 ± 0.05	349.6 ± 7.6	-	2.15 ± 0.03	0.771 ± 0.023	2.80 ± 0.11
med25-2	3.03 ± 0.04	0.980 ± 0.017	1.82 ± 0.04	242.9 ± 3.0	6196 ± 180	2.05 ± 0.05	0.765 ± 0.021	2.24 ± 0.05
ohb1 med25-2	3.15 ± 0.04	0.975 ± 0.014	1.90 ± 0.05	252.5 ± 2.9	6376 ± 210	2.10 ± 0.05	0.755 ± 0.021	2.32 ± 0.02
opr3-1	2.67± 0.03	0.856 ± 0.008	1.37 ± 0.03	202.0 ± 3.1	-	1.75 ± 0.03	0.671 ± 0.019	2.38 ± 0.04
ohb1 opr3-1	2.99 ± 0.04	0.994 ± 0.015	1.83 ± 0.05	240.3 ± 3.2	-	2.06 ± 0.03	0.782 ± 0.022	2.48 ± 0.06
bb-3	2.69± 0.04	0.970 ± 0.016	1.53 ± 0.04	156.6 ± 4.0	-	1.87 ± 0.04	0.803 ± 0.020	2.34 ± 0.02
ohb1 bb-3	3.41 ± 0.06	1.231 ± 0.030	2.56 ± 0.10	211.7 ± 4.2	-	2.34 ± 0.07	0.945 ± 0.032	2.76 ± 0.10
da1-ko1	2.74± 0.04	0.849 ± 0.016	1.43 ± 0.04	156.8 ± 2.7	-	1.86 ± 0.04	0.730 ± 0.026	2.22 ± 0.04
ohb1 da1-ko1	3.42 ± 0.04	1.148 ± 0.022	2.40 ± 0.06	219.3 ± 3.8	-	2.22 ± 0.04	0.878 ± 0.026	2.48 ± 0.04

Each value is expressed as the mean ± SE.
